# Circadian Period Integrates Network Information Through Activation of the BMP Signaling Pathway

**DOI:** 10.1371/journal.pbio.1001733

**Published:** 2013-12-10

**Authors:** Esteban J. Beckwith, E. Axel Gorostiza, Jimena Berni, Carolina Rezával, Agustín Pérez-Santángelo, Alejandro D. Nadra, María Fernanda Ceriani

**Affiliations:** 1 Laboratorio de Genética del Comportamiento, Fundación Instituto Leloir, IIB-BA-CONICET, Buenos Aires, Argentina; 2 Departamento de Química Biológica, Facultad de Ciencias Exactas y Naturales, UBA. IQUIBICEN-CONICET, Buenos Aires, Argentina; Washington University Medical School, United States of America

## Abstract

Living organisms use biological clocks to maintain their internal temporal order and anticipate daily environmental changes. In *Drosophila*, circadian regulation of locomotor behavior is controlled by ∼150 neurons; among them, neurons expressing the PIGMENT DISPERSING FACTOR (PDF) set the period of locomotor behavior under free-running conditions. To date, it remains unclear how individual circadian clusters integrate their activity to assemble a distinctive behavioral output. Here we show that the BONE MORPHOGENETIC PROTEIN (BMP) signaling pathway plays a crucial role in setting the circadian period in PDF neurons in the adult brain. Acute deregulation of BMP signaling causes period lengthening through regulation of d*Clock* transcription, providing evidence for a novel function of this pathway in the adult brain. We propose that coherence in the circadian network arises from integration in PDF neurons of both the pace of the cell-autonomous molecular clock and information derived from circadian-relevant neurons through release of BMP ligands.

## Abstract 2

Circadian pacemaker neurons in the *Drosophila* brain gather network information through the highly conserved BMP signaling pathway to establish the daily period of locomotor behavior.

## Author Summary

The circadian clock controls rhythms in behavior, physiology, and metabolism in all living organisms. The molecular components as well as the neuronal network required to keep this clock running have been identified in several species. In the *Drosophila* brain this neuronal network is represented by an ensemble of 150 neurons, and among them, those expressing the Pigment Dispersing Factor (PDF) neuropeptide encompass the “central oscillator”—also called master clock as it ensures 24-hour periods—of the fly brain. In this study we show that the widely conserved Bone Morphogenetic Protein (BMP) signaling pathway is present in PDF neurons, and upon adult-specific activation it lengthens the endogenous period of locomotor behavior. We find that period lengthening correlates with delayed accumulation of nuclear PERIOD, a core component of the molecular clock. We also identified a putative DNA binding motif for the BMP pathway nuclear components within the regulatory region of the *Clock* (*Clk*) promoter, another core component of the circadian machinery. Interestingly, upon BMP pathway activation endogenous CLK levels are downregulated, thus accounting for the lengthening of the endogenous period. We propose that the endogenous period is a network property commanded by PDF neurons that results from integration of information from both the autonomous molecular clock and the nonautonomous BMP signaling pathway.

## Introduction

Circadian clocks govern many physiological and behavioral changes with an endogenous period of about 24 h and are entrained by environmental inputs such as light and temperature.

The ensemble of biochemical reactions that sustains this phenomenon is well documented and is based on two interconnected negative feedback loops. In the first loop, the clock genes *period* and *timeless* are transcriptionally activated by dCLOCK and CYCLE, giving rise to protein products that negatively regulate their own transcription. In the second loop, dCLOCK and CYCLE activate transcription of their own repressor, VRILLE, and activator PDP1ε. The 24-h period underlying this process is achieved through transcriptional, posttranscriptional, and posttranslational regulation of these core proteins, and by the tight regulation of their subcellular distribution [1]. Through the localization of bonafide clock proteins in the fly brain, over 150 neurons have been ascribed as the circadian neuronal network [2]. Among them, a small bilateral cluster of four neurons called small ventral lateral neurons (sLNvs)—based on their size and relative position in the brain—express the neuropeptide PIGMENT DISPERSING FACTOR (PDF), and several lines of evidence emphasize their relevance as the core pacemaker in *Drosophila* [3]–[6]. This cluster drives morning activity under light–dark cycles (LD) and sets the period in constant darkness (DD) [7],[8]. Clock input pathways for light and temperature have been previously described [9], but the mechanisms in place to define the connectivity within the clock circuit are poorly understood. In particular, how different circadian clusters communicate with each other, the exact nature of their connectivity, and how this information is then transferred to the molecular clock to ultimately produce rhythmic behavioral outputs remains to be determined [10].

Accurate communication among neuronal ensembles is a fundamental process underlying brain function and, in particular, behavioral control. Anterograde communication through synaptic transmission, cell adhesion molecules, or trophic factors allows neurons to signal to postsynaptic targets; in addition, retrograde signaling allows target cells to influence presynaptic neurons. Both types of communication are crucial for neuronal network assembly, maintenance, and function [11],[12].

The *bone morphogenetic protein* (*BMP*) pathway is a highly conserved retrograde signaling pathway that influences synaptic connectivity, ultimately controlling gene transcription. In particular, BMP regulates the growth and strength of the *Drosophila* larval neuromuscular junction (NMJ) [13]–[15]. At the NMJ, the muscle derived ligand GLASS BOTTOM BOAT (GBB) activates the presynaptic motorneuron, which responds by increasing the synaptic size and the amount of neurotransmitter released [16]. In addition, this pathway is crucial in the control of gene expression of circulating neurohormones that modulate NMJ physiology [17],[18]. BMP pathway ligands transmit biological information by binding to type I and type II receptors that form heterotetrameric complexes in the presence of the dimerized ligand and transduce the information to the nucleus through the SMAD proteins [19]. In *Drosophila*, the best characterized orthologs of the BMP type I receptor are *thickveins* (*tkv*) and *saxophone* (*sax*), while *wishful thinking* (*wit*) and *punt* (*put*) account for the only known type II orthologs. Ligand binding links the constitutively active type II receptor kinase to the inactive type I one, which results in the phosphorylation of the latter, thus turning on its kinase activity. As a result, the type I receptor triggers a signaling cascade through phosphorylation of the R-SMAD *Drosophila* ortholog MOTHERS AGAINST DPP (MAD), allowing its association with the Co-SMAD MEDEA (MED). This complex is then translocated to the nucleus to regulate gene expression on its own, or by association with different co-regulators, such as SCHNURRI (SHN) [20],[21]. Following pathway activation, a negative-feedback mechanism is induced through the expression of the I-SMAD DAUGHTERS AGAINST DPP (DAD) [22].

Despite the relevance of retrograde signals for network assembly and maintenance, only one report has focused on a central synapse. Genetic manipulation of the BMP pathway in *Drosophila* motoneurons demonstrated that this activity-dependent retrograde signaling mechanism potentiates synaptic transmission in the central nervous system (CNS) [23].

In this work, we report the identification, through a forward genetic screen, of a new acute regulator of circadian period. Manipulation of gene expression levels demonstrated that the retrograde BMP signaling is necessary for normal circadian behavior. More importantly, pathway activation is crucial for period determination in an adult-specific fashion through the regulation of *Clk* transcription in the sLNvs. Overall, the behavioral and biochemical data presented here indicate that the retrograde BMP pathway acting in the sLNvs modulates the endogenous circadian period, by integrating information from the rest of the circadian network.

## Results

### A Functional Misexpression Screen Uncovers a Role for *schnurri* in Circadian Behavior

**Figure 1:**
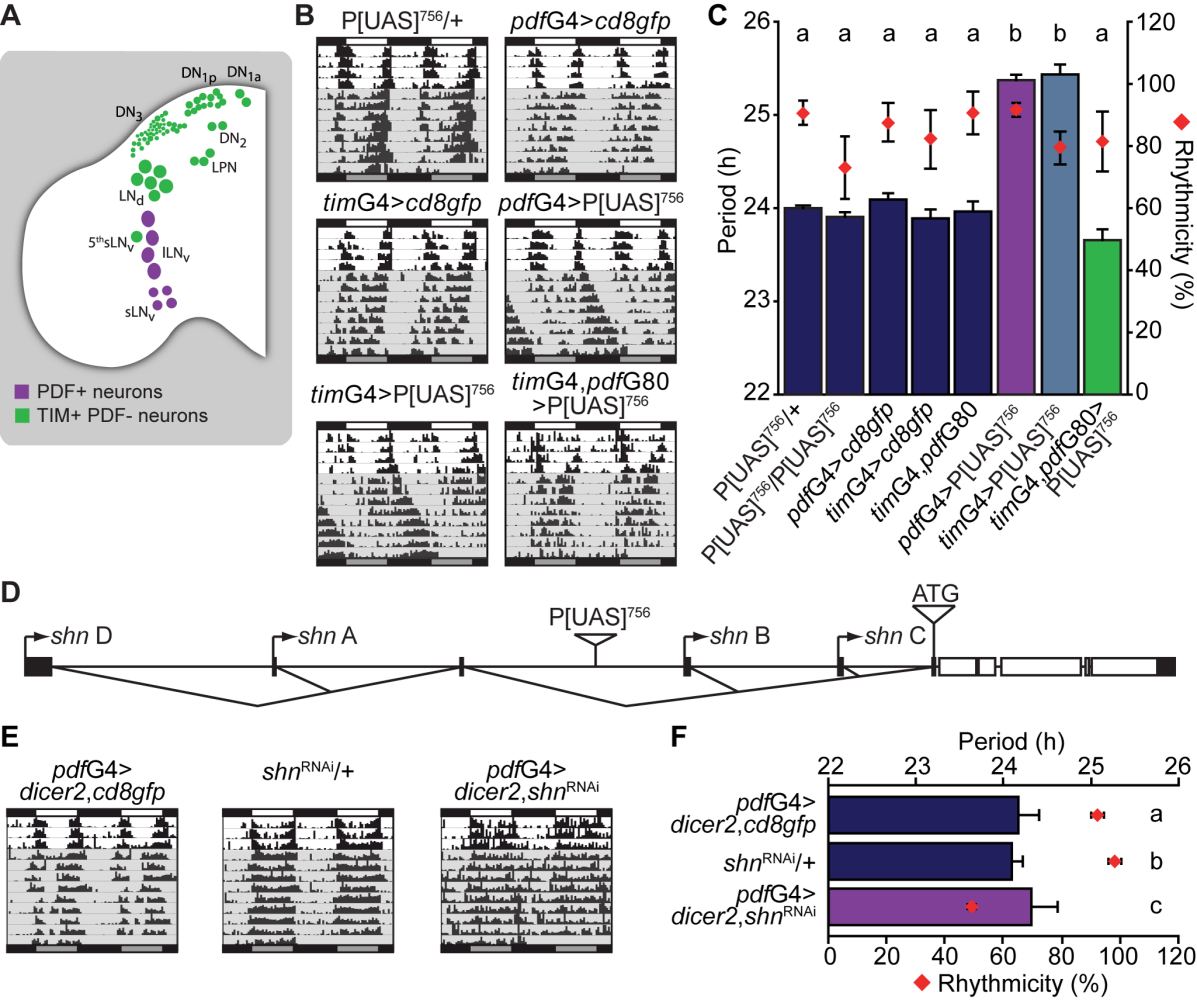
*schnurri* deregulation modulates locomotor behavior in PDF+ cells. (A) Schematic diagram of a fly brain hemisphere displaying all clock-gene expressing neurons. lLNvs, large ventral Lateral Neurons; sLNvs, small ventral Lateral Neurons; LNds, dorsal Lateral Neurons; DN1as, Dorsal Neurons 1 anterior; DN1ps, Dorsal Neurons 1 posterior; DN2s, Dorsal Neurons 2; DN3s, Dorsal Neurons 3; LPNs, lateral posterior neurons. Modified from Muraro et al. [66]. PDF+ and TIM+PDF− neurons were color-coded to facilitate their identification throughout. (B) *shn* overexpression lengthens the endogenous period. Representative double-plotted actograms of individual flies of the indicated genotypes. During the experiments, flies were kept in LD for 4 d, then switched to DD (shaded gray area), and monitored for 8 additional days. (C) Graph shows the quantitation of period and rhythmicity for the indicated genotypes; bars and diamonds represent average period and percentage of rhythmicity, respectively. Statistical analysis included one-way ANOVA for period determination, and different letters indicate significant differences in Tukey comparisons, α = 0.05. Purple bars indicate the treatments in which the BMP pathway is being modulated in PDF+ neurons. (D) Schematic diagram of the *shn* locus; the four alternative transcription initiation sites, the relative position of the transposon, and the common translation initiation site (ATG) are indicated (not to scale). Black boxes, untranslated regions; white boxes, coding region. (E) *shn* knockdown leads to deconsolidation of locomotor activity. Representative double-plotted actograms of individual flies of the indicated genotypes. During the experiments, flies were kept in LD for 3 d, then switched to DD (shaded gray area), and monitored for 9 additional days. (F) Graph shows the quantitation of period and rhythmicity for the indicated genotypes (see legend to Figure 1C for more details). Statistical analysis for rhythmicity data included one-way ANOVA, and different letters indicate significant differences in Tukey comparisons, α = 0.05. Error bars represent SEM and averages of at least three independent experiments. See Tables S1 and S2 for details.

Great efforts have been dedicated to uncover new genes involved in the modulation of circadian rhythms. To identify additional components involved in sending or receiving information relevant for synchronization of the circadian network, a misexpression screen was carried out through deregulation of gene expression specifically in PDF expressing (PDF+) cells (Figure 1A). The *pdf*Gal4 (herein referred to as *pdf*G4) transgenic fly [5] was employed to drive expression of independent transgenic insertions generated in our laboratory from a founder P[UAS] line [24],[25]. As a result, we identified a fly strain, P[UAS]^756^, that causes period lengthening of daily activity rhythms (Figure 1B and 1C). The transposon landed within *schnurri* (Figure 1D), a zinc finger protein that acts as a partner of SMADs, and is crucial for repression of target genes upon activation of the BMP pathway [20],[21],[26]–[28]. Interestingly, misexpression of *shn* in the entire circadian circuit through *tim*G4 [29] led to a similar long period phenotype. However, addition of *pdf*G80—that prevents GAL4 function in PDF+ cells [8]—rescued the endogenous period in *tim*G4>P[UAS]^756^ flies (Figure 1B and 1C), indicating that PDF+ cells constitute a crucial cluster to transduce the BMP signal and modulate the locomotor activity period.

Based on the initial *in silico* analysis it was envisioned that P[UAS]^756^ could mediate overexpression of the *shn* locus upon GAL4 activation. To test this, we performed qPCR analysis to measure the four alternative transcripts encoded by this locus according to Flybase (Figure S1A). No effect on overall levels of *shn* were observed in homozygous P[UAS]^756^ flies (Figure S1B). However, when P[UAS]^756^ was acutely induced in the entire organism, a significant increase was specifically observed in variant B, whose start site is located immediately downstream of the P[UAS]^756^ landing site (Figure S1A and S1C). These results suggest that increased levels of *shn* are responsible for the behavioral phenotype observed. In support of this, overexpression of *shn* in the PDF neurons through an independent UAS line [30] also led to a long period phenotype, similar to that observed in *pdf*G4>P[UAS]^756^ flies (Figure S1D and S1E). In addition, we rescued the P[UAS]^756^ overexpression phenotype through the concomitant expression of a specific *shn* ^RNAi^ predicted to target all four alternative transcripts (Figure S1F and S1G).

To explore an intrinsic effect of *shn* within the circuit we used the *pdf*G4 strain to drive *shn* ^RNAi^ in the LNvs; this treatment renders only 50% of rhythmic flies, with a clear disorganization of the activity profile, pointing to a specific function of *shn* on locomotor rhythms (Figure 1E and 1F). A potential role for *shn* in the sLNv cluster is further supported by the observation that there is an enrichment of *shn* mRNA in PDF+ relative to ELAV+ neurons [31].

### The BMP Signaling Cascade Is Active Within the sLNvs

**Figure 2:**
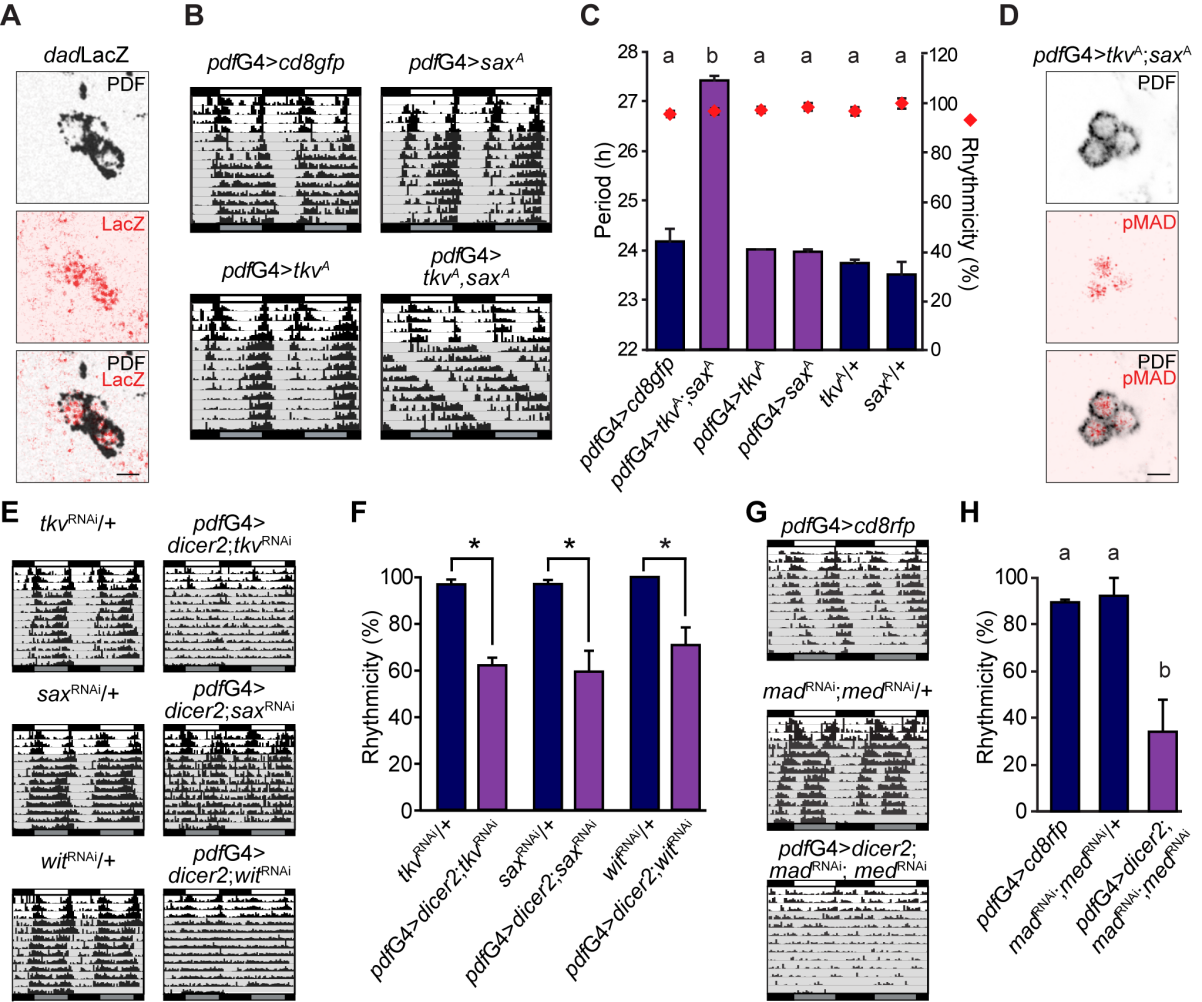
The BMP pathway is active in adult core pacemaker neurons. (A) The BMP target gene *dad* is expressed in the sLNvs. Brains dissected from *dad*LacZ reporter flies were immunostained with anti-PDF (black) and anti-LacZ (red) antibodies at ZT 02. Images correspond to the sLNvs. Scale bar, 5 µm. (B) Concomitant expression of *tkv* ^A^ and *sax* ^A^ slows the pace of the clock. Representative double-plotted actograms of flies of the indicated genotypes. Behavioral experiments were carried out as detailed in the legend to Figure 1E. (C) Graph shows the quantitation of period and rhythmicity for the indicated genotypes (see legend to Figure 1C for more details). Period data were analyzed with one-way ANOVA, and different letters indicate significant differences in Tukey comparisons, α = 0.05. (D) *mad* is expressed in pacemaker neurons. The sLNvs from an adult brain expressing both *tkv* ^A^ and *sax* ^A^ stained for anti-PDF and anti-pMad are shown. Scale bar, 5 µm. (E) Constitutive down-regulation of type I receptors results in deconsolidated activity. Representative double-plotted actograms of flies of the indicated genotypes. Behavioral experiments were carried out as detailed in the legend to Figure 1E. (F) Percentage of rhythmicity for each indicated genotype, * *p*<0.05 (Student's *t* test). (G) Joint *mad* and *med* down-regulation induces strong arrhythmicity. Representative double-plotted actograms of flies of the indicated genotypes. Behavioral experiments were carried out as detailed in the legend to Figure 1E. (H) The graph shows the quantitation of rhythmicity for the indicated genotypes. Data were analyzed with one-way ANOVA, and different letters indicate significant differences in Tukey comparisons, α = 0.05. Error bars represent SEM and averages describe a minimum of three independent experiments. See Tables S1 and S2 for details.

Since *shn* is a nuclear component of the BMP canonical pathway [20], we decided to evaluate the involvement of the entire pathway in the control of circadian locomotor activity. We first checked whether the pathway is active within the PDF cells employing a well-characterized reporter line (*dad*LacZ [22]). This strategy showed a clear activation in PDF+ cells (Figure 2A and Figure S2A) and in some of the DN1 neurons (Figure S2B), while it is clearly absent from other clusters—that is, the LNd neurons (Figure S2A). Subsequently we expressed constitutively activated forms of the type I receptors *sax* and *tkv* (*sax^A^* and *tkv^A^*, respectively) [32],[33] in the PDF neurons. Despite *sax^A^* or *tkv^A^* expression failed to modulate behavior on their own, when expressed together they led to a surprisingly long period phenotype, almost 2 h longer than the deregulation of the downstream nuclear effector (Figure 2B and 2C), a condition that allowed detection of a clear pMad signal in the nucleus of the sLNvs (Figure 2D and Figure S3). However, it was not possible to detect this marker of pathway activation in a wild-type brain (Figure S3A), likely due to low levels. The fact that joint *tkv* and *sax* up-regulation leads to a long period phenotype is in agreement with the change observed upon *shn* overexpression, since *shn* and the receptors are positive elements of the signaling pathway.

Taking into account that expression of activated receptors could result in an ectopic phenotype, we down-regulated the endogenous receptors through specific RNAi lines in a cell-autonomous fashion. Interestingly, reduced levels of distinct type I and II receptors in PDF neurons resulted in deconsolidation of the activity pattern leading to arrhythmicity (Figure 2E and 2F), phenocopying *shn* down-regulation (Figure 1E and 1F). In agreement with these findings, joint down-regulation of *mad* and *med*, the nuclear components of the signaling pathway, produces an even more severe effect on rhythmicity (Figure 2G and 2H). In addition to the partial decrease in rhythmicity, down-regulated expression of BMP pathway components reduced the strength of locomotor rhythmicity in most flies, even the ones initially scored as rhythmic (Table S2).

In summary, these findings support the notion that the BMP pathway operates within PDF neurons and contributes to the control of locomotor activity rhythms.

### A Blend of BMP Ligands Shapes Locomotor Activity Rhythms

**Figure 3:**
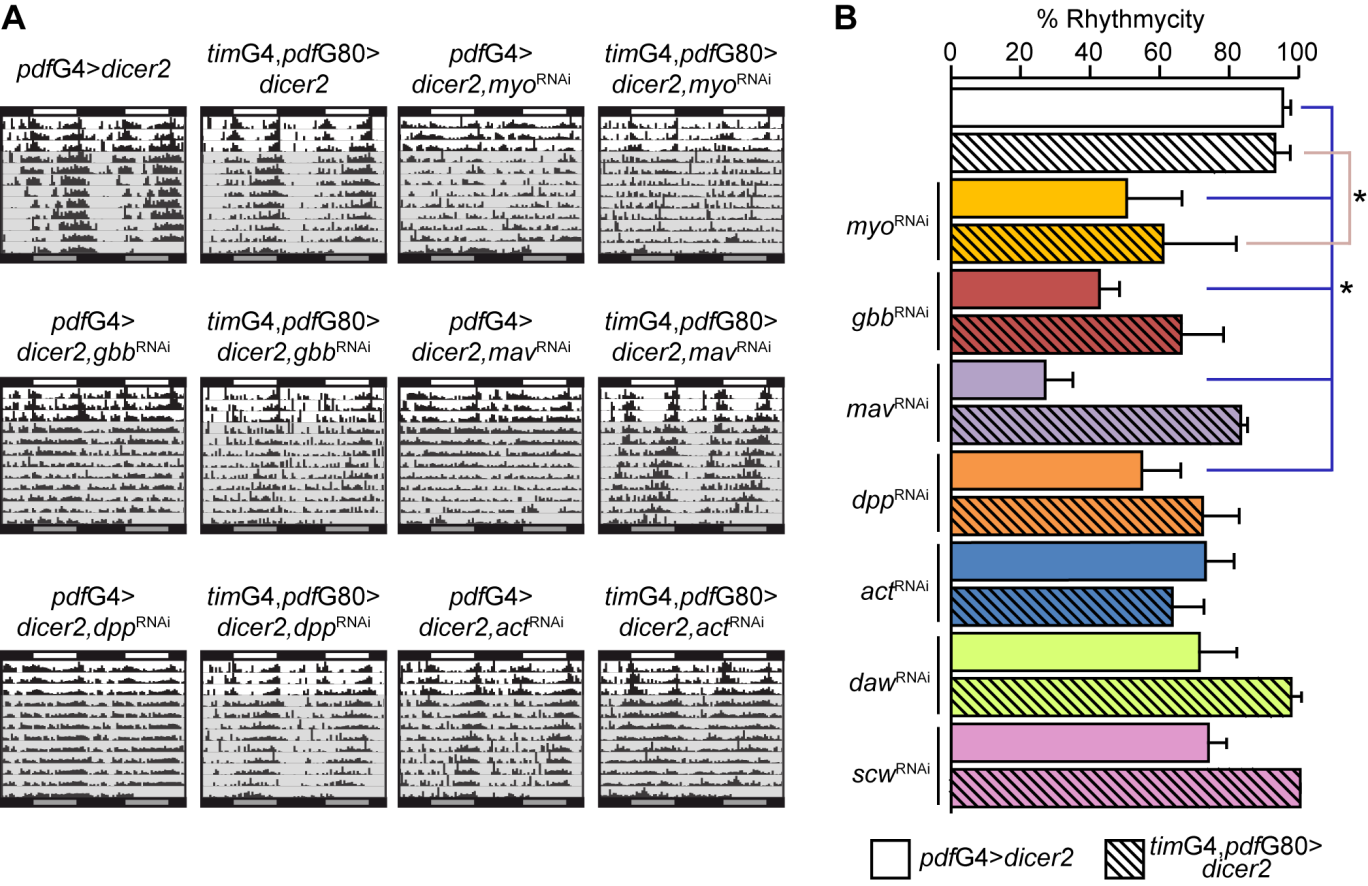
A combination of BMP ligands orchestrate the function of the circadian network. (A) Representative double-plotted actograms of flies of the indicated genotypes. Behavioral experiments were carried out as detailed in the legend to Figure 1E. (B) Graph shows the quantitation of rhythmicity for the indicated genotypes. Statistical analysis included one-way ANOVA; * indicates significantly different treatments in Tukey comparisons, α = 0.05. Error bars represent SEM and averages represent at least three independent experiments. See Table S3 for details.

The finding that the BMP pathway operates in the sLNvs prompted us to identify which of the seven members of the ligand superfamily [19] are critical for the control of locomotor rhythms. Since this signaling pathway functions retrogradely, we reasoned that a putative ligand source could either be the TIM+PDF− neurons or the PDF+ lLNvs (Figure 1A). Thus, we down-regulated specific ligands either in PDF+ or TIM+PDF− neurons by means of RNAi expression. Interestingly, several ligands affected rhythmicity (Figure 3A and 3B). Although *myoglinin* (*myo*) appears to play a role in both clusters, *gbb*, *maverick* (*mav*), and *decapentaplegic* (*dpp*) expression is relevant for rhythmycity in PDF+ cells. On the other hand, down-regulation of either *activin-β* (*actβ*), *screw* (*scw*), or *dawdle* (*daw*) did not affect rhythmicity on either cluster (Figure 3A and 3B), although these RNAi lines were able to trigger lethality when driven from a constitutive promoter (such as *actin*; unpublished data). However, a more detailed analysis addressing the strength of the rhythmicity indicates that with the exception of *dawdle*, all ligands affect to some extent the consolidation of the activity pattern in either cluster (Table S3). These observations open the attractive possibility that different neuronal clusters release several BMP ligands to communicate with PDF neurons.

Despite several lines of evidence supporting a role for this signaling pathway in fine-tuning circadian activity rhythms (Figures 1–3), the contribution to the phenotype of miss-expression throughout development of distinct pathway components cannot completely be ruled out.

### Adult-Specific *shn* Overexpression Triggers a Long Period Phenotype

The activation of the BMP pathway throughout development could lead to structural abnormalities [16], in turn affecting behavior. To discard potential developmental effects on period determination, we employed the temperature inducible TARGET system [34] to acutely activate *shn* overexpression in the adult circadian circuit.

**Figure 4:**
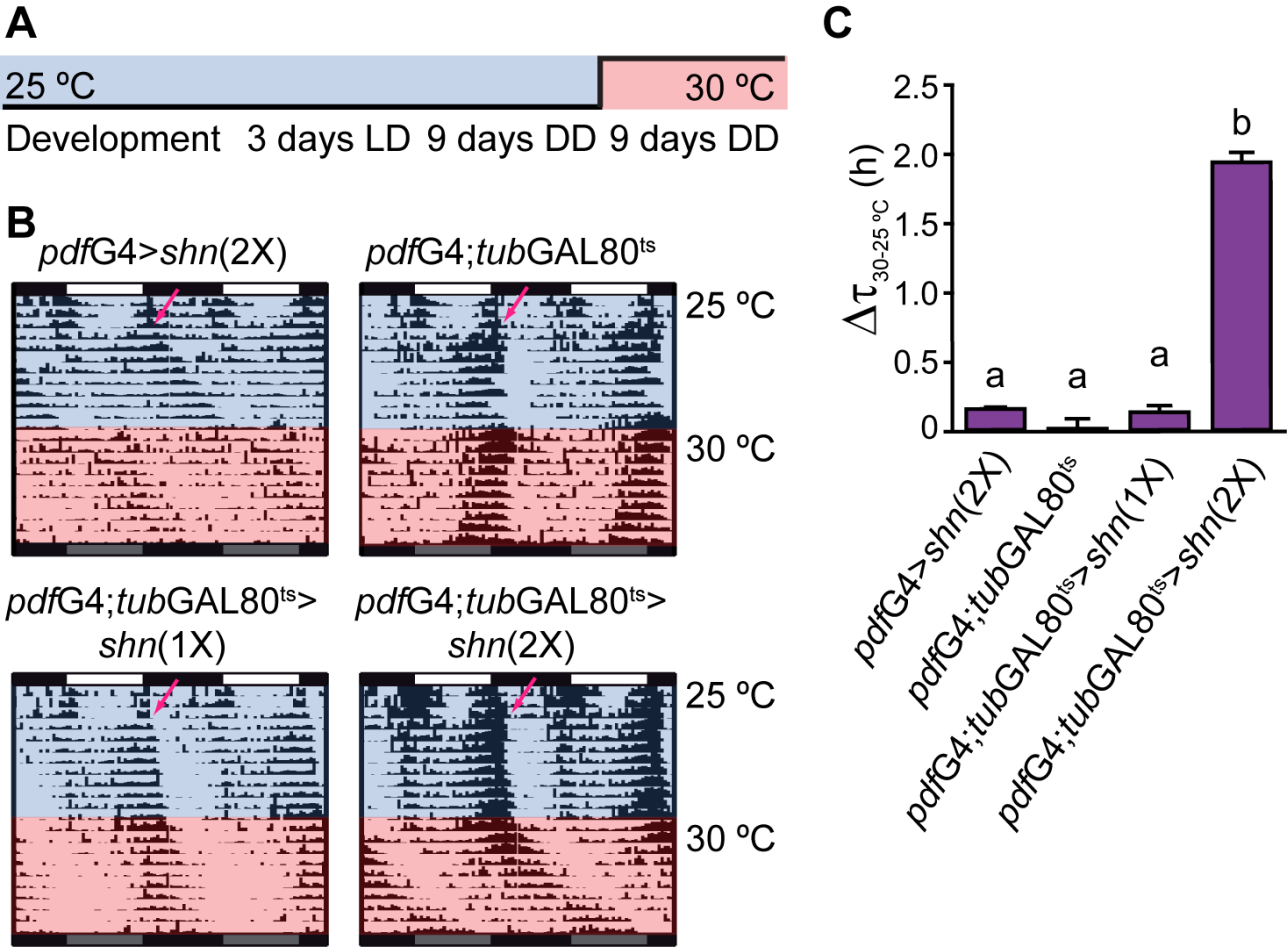
*schnurri* acutely modulates behavior in adult flies. (A) Schematic diagram of the experiment shown in Figure 3B and 3C; flies were raised at 25°C under LD cycles; 0–3-d-old flies were loaded activity monitors and entrained for 3 d in LD, and then transferred to DD. After 9 d in DD (at 25°C) the flies were transferred to fresh test tubes and kept at 30°C (permissive temperature) in constant darkness for additional 9 d. (B) Representative double-plotted actograms of flies of the indicated genotypes. The blue and red shadows represent the phase at the restricted (25°C) and permissive (30°C) temperature, respectively. Arrows indicate the transfer to DD. (C) Bar diagram shows the difference between the endogenous period at the permissive and restrictive temperatures for each specific genotype. Data were analyzed with one-way ANOVA, and different letters indicate significant differences in Tukey comparisons, α = 0.05.

Flies were raised and tested during the first part of the behavioral experiment at the restrictive temperature (preventing GAL4 activity), and then were transferred to the permissive temperature (allowing GAL4 activity); thus, locomotor behavior was recorded in a condition that allows acute *shn* overexpression (Figure 4A). Despite the temperature shift, control flies—as well as those carrying a single transgenic copy of *shn*—retained their endogenous period. Along the same line, constitutive expression of *shn* in PDF neurons was subtly affected by the temperature increase. In contrast, flies carrying two copies of the *shn* transgene showed a significant period lengthening of 2 h, which became evident immediately after the temperature shift (Figure 4B and 4C). Therefore, the fact that acute *shn* overexpression results in a period-lengthening phenotype demonstrates that this pathway impacts the endogenous period in clock neurons, likely in response to a retrograde signal.

To rule out structural defects associated to *shn* manipulation restricted to adulthood, we evaluated circuit morphology in flies induced for 2 or 9 d as adults (Figure S4A). A membrane-tethered version of the red fluorescent protein (CD8RFP) was employed to more precisely describe the entire PDF arborizations. No differences were detected between groups subjected to 2 or 9 d of *shn* overexpression in adults (Figure S4B). We specifically analyzed dorsal projections from sLNvs given their relevance in circadian control [35]; no difference either in their morphology or their length was detected (Figure S4C and S4D). The addition of an extra UAS-driven transcript describing the entire circuitry did not reduce the strength of the original behavioral phenotype (Figure S4E–G).

The absence of gross structural defects associated to *shn* misexpression reinforces the notion of an acute role of the BMP pathway in setting the pace of the molecular clock.

### Delayed PER Entry to the Nucleus Correlates with the Behavioral Phenotype

**Figure 5:**
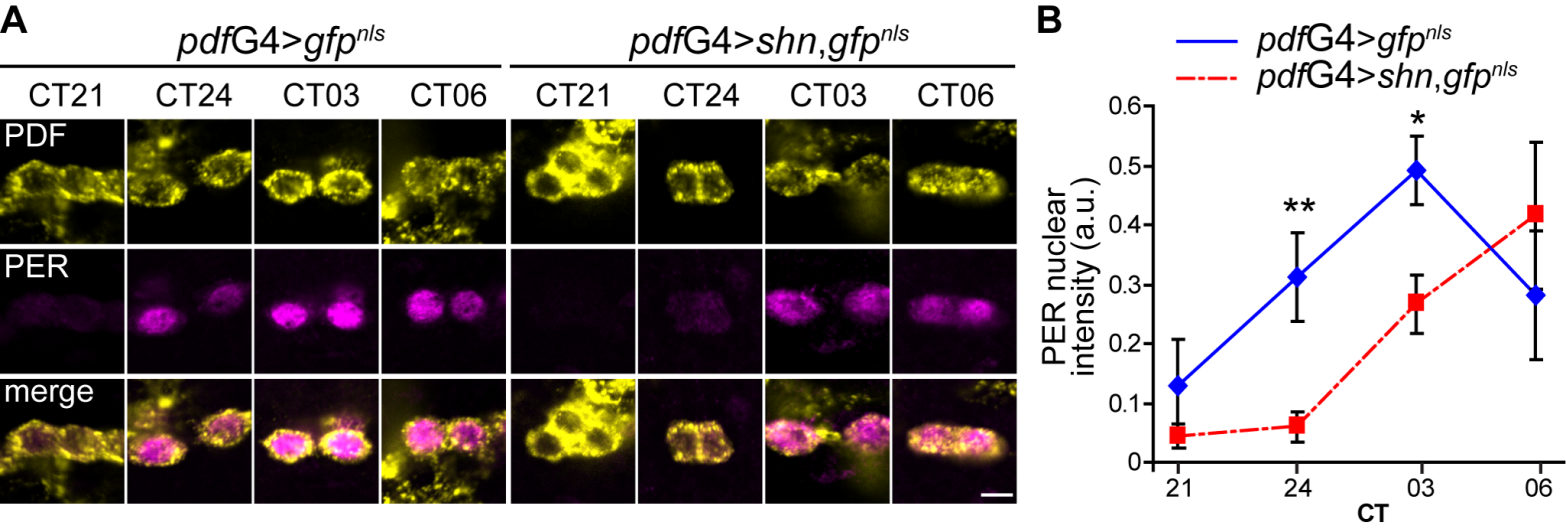
PER accumulation and nuclear entry are delayed in *shn* overexpressing sLNvs. (A) Whole mount brain immunofluorescence was performed to follow PDF (upper panel) and PER (middle) accumulation during the transition between DD3 and DD4. Representative single plane confocal images of sLNvs for the indicated time points and genotypes are shown. Images were taken employing the same confocal settings throughout an individual time course. The experiment was repeated three times with similar results. Scale bar, 5 µm. (B) Quantitation of PER nuclear intensity. The nuclear area was delimited using the GFP^NLS^ signal, so that PER immunoreactivity quantitation was restricted to that area; the PDF positive staining and cell body size was used to identify sLNvs. Between 9 and 10 brains were analyzed per time point; the average of 2–4 neurons was used for each determination. Three independent experiments were analyzed with two-way ANOVA; the interaction between factors was significant and simple effects were analyzed comparing genotypes, CT24 *p* = 0.0452 (**) and CT03 *p = *0,067 (*).

The oscillation of PER protein abundance and subcellular localization is a hallmark of the molecular clock, and its nuclear translocation is a crucial step in this process [36]–[39]. To confirm that the period lengthening defect that derives from *shn* overexpression correlates with the pace of the molecular oscillations of core clock components, PER immunoreactivity was assessed in sLNvs during the transition between the third and fourth day in constant darkness. As expected, PER nuclear translocation was delayed; while in control brains PER could be detected in the nucleus by CT24 with a maximum at CT03, in *pdf*G4>*shn* PER is almost absent from the nucleus by CT24 (Figure 5A). Interestingly, PER accumulation in sLNvs initiated at CT03 in *pdf*G4>*shn* flies, and was still detectable in the cytoplasm by CT06 (Figure 5A and 5B). Although PER accumulation was delayed, the overall levels were not grossly affected (Figure 5B).

**Figure 6:**
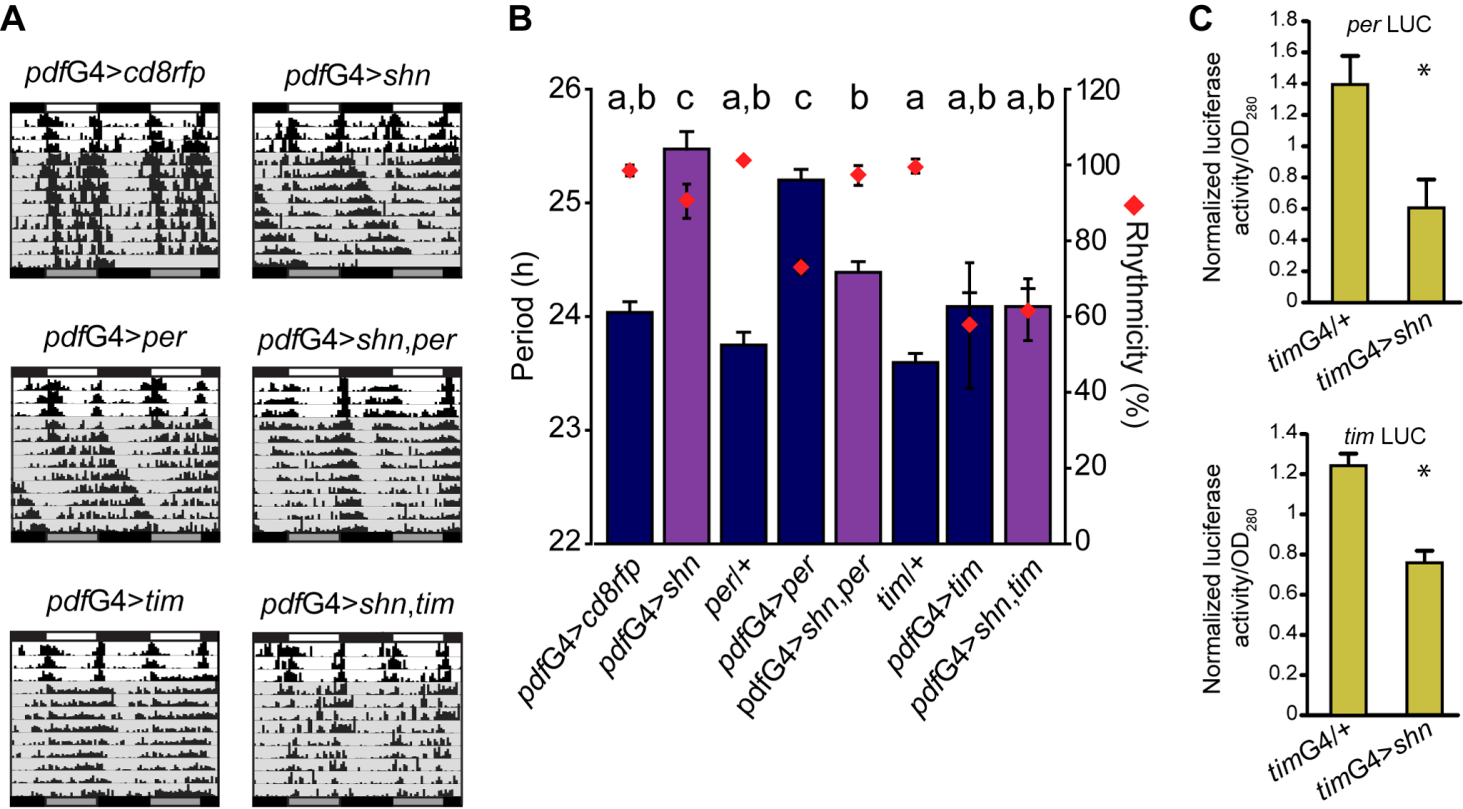
Increased PER and TIM levels rescue the behavioral phenotype associated with *shn* overexpression. (A) Representative double-plotted actograms of flies of the indicated genotypes. Behavioral experiments were carried out as detailed in the legend to Figure 1E. (B) Graph shows the quantitation of period and rhythmicity for the indicated genotypes (see legend to Figure 1C for more details). Statistical analysis included one-way ANOVA for period determination, and different letters indicate significant differences in Tukey comparisons, α = 0.05. Error bars represent SEM, and averages represent at least three independent experiments. See Table S1 for details. (C) *per* and *tim* are transcriptionally modulated by *shn*. Luciferase activity from total head extracts was measured at ZT02 in transgenic flies carrying *per* (upper panel) or *tim* (bottom panel) transcriptional reporters combined with *shn* overexpression in the entire clock circuit (*tim*-G4). Three independent experiments were carried out. Data from each experiment were normalized against the mean value of all measurements to contemplate different absolute luciferase activity levels. Three independent experiments were carried out and were analyzed with Student's *t* test; *per* LUC *p* = 0.020, *tim* LUC *p* = 0.002.

Activation of the BMP pathway leads to transcriptional regulation, in which SHN plays a relevant role [21],[26],[40]. To test the idea that the behavioral phenotype derives from a delay in *per* transcription and the subsequent lag in protein accumulation, we assayed the effect of expressing *per* in the context of elevated SHN levels. As it was formerly reported, PER overexpression (by means of a UAS-*per* transgene) led to a long period phenotype (Figure 6A,B and [41]), likely through constantly high PER levels driven by a constitutive promoter. Surprisingly, overexpression of both transgenes (*shn* and *per*) resulted in highly rhythmic flies, with periods close to 24 h (Figure 6A and 6B). Similar results were observed when *shn* and *tim* were simultaneously expressed (Figure 6A and 6B).

To rule out the possibility that the presence of additional UAS-driven transgenes could account for the behavioral rescue (i.e., resulting in the dilution of the original long period phenotype), we introduced one or two unrelated UAS-driven transgenes in the *pdf*G4>*shn* background. These manipulations failed to alter the original phenotype (Figure S5A and S5B), reinforcing the specificity of the PER and TIM rescues.

To further support the idea that the BMP pathway is impinging upon *per* and *tim* promoters, we resorted to the use of luciferase reporters for both genes. s*hn* overexpression in the entire circadian network showed a clear reduction of *per* and *tim* transcription (Figure 6C).

These observations clearly underscore that the BMP pathway modulates at the transcriptional level these two core clock genes.

### SHN Impinges Upon *Clk* Promoter Activity

**Figure 7:**
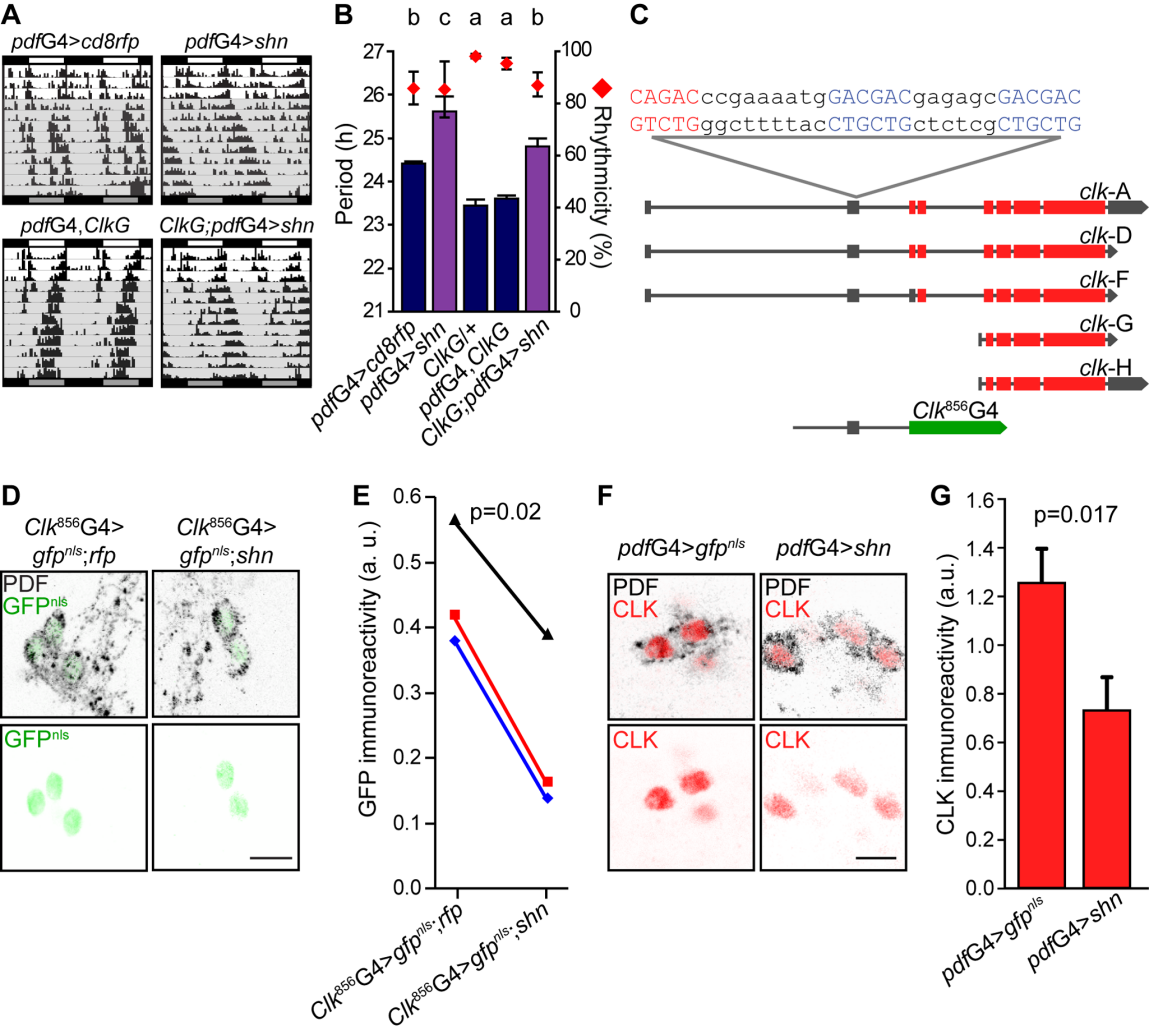
SHN down-regulates CLK protein levels through modulation of *Clk* promoter activity. (A) Representative double-plotted actograms of flies of the indicated genotypes. Behavioral experiments were carried out as detailed in the legend to Figure 1E. (B) Graph shows the quantitation of period and rhythmicity for the indicated genotypes (see legend to Figure 1C for more details). Statistical analysis included one-way ANOVA for period determination, and different letters indicate significant differences in Tukey comparisons, α = 0.05. Error bars represent SEM, and averages represent at least three independent experiments. See Table S1 for details. (C) The schematic diagram depicts the *Clk* locus highlighting the existence of five alternative transcripts (according to FlyBase) along with the DNA fragment contained in the GAL4 reporter line; gray boxes represent untranslated exons, red boxes indicate translated exons, and the green box indicates the fusion between the first 18 amino acids of CLK and the transcription factor GAL4 (not to scale). Putative MAD (red) and MED (blue) binding elements and the relative position within the locus are indicated. The Clk^856^GAL4 reporter line contains a total of 2,334 bp [51]. (D) Whole mount brain immunofluorescence was performed to follow PDF (upper panel) and GFP (upper and bottom panels) accumulation at ZT2 in the −856 *Clk* reporter line (*Clk* ^856^). This time point was selected to reduce the impact of differences stemming from the period lengthening phenotype. Representative confocal images of sLNvs for the indicated genotypes are shown. Note that controls also include a second UAS-driven transgene. Scale bar, 5 µm. (E) Quantitation of GFP nuclear intensity. PDF+ staining and cell body size were used to identify sLNvs. A total of 8 to 10 brains were analyzed in each experiment; the average of 2–4 neurons was used for each determination, and the images were taken employing the same confocal settings throughout an individual experiment. Three independent experiments were carried out, and data were analyzed with Randomized Blocks ANOVA to contemplate potential differences due to the different confocal settings. Paired measurements are linked by colored lines. p_blocks_ = 0.04, p_genotypes_ = 0.02. (F) Whole mount brain immunofluorescence was performed to follow PDF (upper panel) and CLK (upper and bottom panels) accumulation at ZT14 in the indicated genotypes. This time point was selected to maximize CLK levels and reduce the impact of the period lengthening phenotype. Scale bar, 5 µm. (G) Quantitation of CLK nuclear intensity, as described in (E). Five independent experiments were carried out, and data were analyzed with Student's *t* test, *p* = 0.017.

It is well established that CLOCK is the main *per* and *tim* transcriptional activator [42]; therefore, the period lengthening phenotype could arise from decreased CLK activity in *pdf*G4>*shn* flies. Given *shn*'s role as a negative regulator of transcription, we sought to determine if an increase in *Clk* gene dosage could modulate the *shn*-dependent behavioral phenotype [43]. Interestingly, the presence of an additional copy of the *Clk* locus (*ClkG*) partially rescued the long period phenotype derived from *shn* overexpression (Figure 7A and 7B). In a complementary approach, we also expressed *Clk* by means of a UAS-*Clk* transgene in the context of *shn* overexpression. As reported, elevated CLK levels make no impact on the free-running period of locomotor behavior, although the original UAS line *per se* subtly shortens the period (Figure S6A,B and [44]). In favor of the possibility that *shn* overexpression could directly or indirectly lead to reduced CLK levels, *pdf*G4>*shn*,*Clk* flies exhibited an intermediate phenotype (shorter than *pdf*G4>*shn* and longer than *pdf*G4>*Clk*; Figure S6A and S6B), suggesting that, as it is the case for *per* and *tim* expression, *Clk* is able to rescue the period-lengthening phenotype. Together, these results suggest that *Clk* is likely targeted once the pathway is activated. Using previously characterized DNA-responding elements for the BMP pathway [21],[45]–[49] we searched for putative DNA binding sites within the *Clk* promoter. Interestingly, we found two MAD binding sites in close relationship with one MED site, constituting a potential regulatory element for the BMP pathway in the *Clk* promoter (Figure 7C).

To further address whether SHN modulates *Clk* transcription specifically in the sLNvs, we resorted to a reporter assay based on GFP^nls^ immunoreactivity [50]. Joint expression of GFP^nls^ and SHN driven by the *Clk* reporter line *Clk^6939^* (Figure S6C and [51]) resulted in clearly reduced GFP accumulation when compared to controls (Figure S6D and S6E). Furthermore, a similar trend was observed when employing a minimal *Clk* promoter present in the reporter line *Clk^856^* (Figure 7D and 7E), which also contains the putative DNA responding elements (Figure 7C). To establish that *shn* overexpression leads to a reduction in *Clk* levels, we examined CLK inmunoreactivity in the nuclei of sLNvs at ZT14. In line with our previous observations, CLK levels were reduced by 40% (Figure 7F and G), further confirming that *shn* overexpression reduces *Clk* promoter activity.

In sum, both behavioral and transcriptional approaches underscore a role for the BMP pathway in modulating *Clk* transcription, which in time impacts on *per* and *tim* transcription and ultimately on the pace of the molecular clock.

## Discussion

To identify molecules relevant to the control of rhythmic behavior, a genetic screen was carried out through deregulation of gene expression in core pacemaker cells. As a result, a fly strain that causes period lengthening of daily activity rhythms was singled out, which pointed to a nuclear component of the BMP pathway. Adult-restricted activation of this signaling pathway in the sLNvs led to an increase in the endogenous free-running period, while RNAi-mediated knock-down of specific pathway components highlighted their requirement in coherent behavior. At a molecular level we demonstrated the presence of pathway components in specific subsets of circadian neurons and that pathway activation triggers a delayed nuclear PER accumulation through the negative regulation of the *Clk* promoter. Together, these findings provide strong evidence that the BMP signaling is present in the sLNvs and modulates the pace of the clock, providing a fine-tuning mechanism for a network-dependent setting of the circadian period.

### Adult-Specific BMP Pathway Activity in the CNS

Retrograde signaling is a conserved mechanism regulating neuronal development and function through determination of transmitter phenotype, transcription factor profiles, network connectivity, and synaptic efficacy [11],[16]. However, the essential nature of the processes governed by BMP signaling has precluded a comprehensive analysis in the adult fly. In this study we demonstrated that this pathway is present in the adult fly brain and we defined an acute role for it (Figures 2, 3, 4, S2, and S3). The ability of *shn* overexpression to slow the pace of the clock during adulthood is the first evidence, to our knowledge, of a nondevelopmental behavioral function of the BMP pathway in an intact adult organism.

A well-established role for the BMP retrograde pathway is the ability to determine neuronal identity through modulation of gene expression. Expression of the neuropeptide FMRFamide in neurosecretory Tv neurons is completely abolished in *wit* mutants [52]. BMP retrograde signal is required early on to determine their peptidergic identity [53], and then to maintain the expression of this neuropeptide in the adult brain [18]. Similar results were observed in Crustacean Cardioactive Peptide (CCAP) neurons [17], a subset of neurosecretory cells that control the behavioral program underlying ecdysis [54]. This data prompted us to evaluate the role of pathway deregulation on PDF expression, although reduced *pdf* levels result in a shortening of the free-running period [55]. Neither *pdf* transcriptional levels nor PDF neuropeptide accumulation at the dorsal sLNvs terminals were affected upon *shn* overexpression in the adult (unpublished data). Recent work from our laboratory demonstrated that the concerted action of the BMP and PDF signaling pathways is required early on during development to define the adult architecture of PDF neurons [56]. However, the fact that BMP signaling is active in the adult to modulate period length, with no effect on circuit morphology (Figure S4), rules out the possibility that period lengthening derives from altered PDF levels. These observations suggest a different role for the BMP pathway in core pacemaker cells in the adult brain.

### Different Components of the BMP Pathway Trigger Circadian Phenotypes

RNA interference-mediated knockdown enabled us to test the involvement of the BMP type I and II receptors, ligands, as well as the nuclear components in circadian control of locomotor behavior. Reduced BMP components result in a mutant phenotype, characterized by a disorganized locomotor activity profile (Figures 2 and 3), with no effect on period. Although more experiments are ensured to properly address this difference, the pleiotropic nature of this signaling pathway and the genetic strategies employed (including expression throughout development) could account for the behavioral differences.

In light of the well-known role of this pathway as a retrograde signal in neuronal communication, the fact that down-regulation of any pathway component results in deconsolidation of rhythmic activity opens the possibility that this mechanism is recruited for the coordination of the neuronal network underlying circadian locomotion. The different penetrance of the behavioral phenotypes among pathway components could be accounted for either a technical issue regarding the efficacy of specific UAS-driven RNAi lines, the differential strength of particular drivers (i.e., resulting from the *pdf* and *tim* promoters), or to the relevance that each molecule plays in the underlying process. As an example, different receptors (whose reduction leads to 60% of rhythmicity; Figure 2F) could partially replace each other, while the concomitant down-regulation of both *mad* and *med* (which renders only 30% of rhythmic animals; Figure 2H) argues in favor of a mandatory role for these molecules in the coordination of rhythmic locomotion.

**Figure 8:**
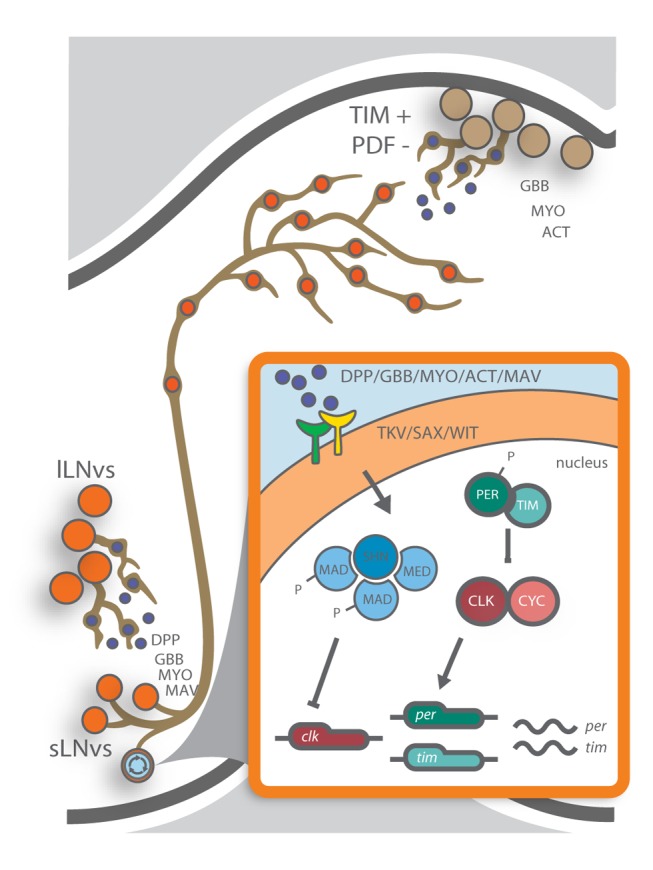
Retrograde signaling impacts period determination. The figure depicts a model in which different ligands released from sLNv postsynaptic targets (TIM+PDF− as well as the lLNvs) modulate the activity of the BMP signaling cascade in the sLNvs acting through the TKV/SAX/WIT receptors. In turn, this signal results in a transcriptionally active complex (including *schnurri*) that down-regulates *Clk* expression. Reduced *Clock* levels would then give rise to decreased *per* transcription and therefore reduced PER levels, thus slowing the pace of the molecular oscillator.

An alternative strategy extensively used to study BMP signaling is the expression of constitutively active forms of the *sax* or *tkv* type I receptors [20]. The longer period phenotype observed upon receptor activation compared to the one achieved through *shn* deregulation suggests that period length correlates with the degree of BMP activation in the sLNvs. In addition, the observation that two distinct type I receptors are necessary for the period lengthening phenotype suggests a complex combination of BMP ligands reaching the PDF neurons (Figure 2 and [56]). The notion that two type I receptors need to be activated to modulate the circadian system is further supported by the correlation between the period phenotype and the detection of pMAD immunoreactivity in the nucleus of the sLNvs (Figure S3). While SAX activation leads to nondetectable pMAD levels, TKV activation associates to moderate levels of nuclear pMAD; instead, activation of both receptors leads to significantly higher pMAD levels in the sLNvs and in turn triggers a profound change in the endogenous period (Figure S3B). It has previously been described that in certain developmental processes the combination of ligands and receptors is important for the proper function of the cascade, thus broadening its flexibility [32]. In the case of the sLNvs, our findings open the possibility that different postsynaptic targets could release specific ligands, in turn enriching the communication between the clusters of the circadian network (Figure 8). In addition, constitutively active receptors allowed us to show that upon activation pMad is present in the nucleus of the sLNvs (Figures 2A and S3). In this context it is worth noting that we performed a time course in a wild-type background looking for differential pathway activation along the day, but we could not detect the phosphorylated form of this protein at any time point (unpublished data). This inability to detect pMAD could merely reflect the fact that either the time resolution (a 6-h window) was insufficient, or this signal is transient (short lived). A potential transient effect of this signaling pathway could result from ligands released from the postsynaptic targets of the sLNvs that could vary in response to a variety of stimuli (internal or external) in order to balance network function and achieve coherence within neuronal clusters. In this context it is likely that subtle or transient increases in pMAD levels (undetectable through immunohistochemistry) could still support a proper pathway function in the sLNvs in a wild-type brain.

An interesting turn to the relationship between the BMP pathway and the molecular clock came from the finding that its effector, MAD, was identified in a high throughput analysis as a CLK target gene [57], implying the possibility of a negative feedback loop between these two transcription factors.

### Retrograde BMP Signal Fine-Tunes Circadian Transcription

Manipulation of the dosage of core clock genes has provided a reliable strategy to unravel the mechanisms leading to period determination, since any period effect resulting from a change in gene dosage likely points to a rate-limiting step [58]. In this regard, the manipulation of *per* dosage has clearly paved the way. An increase in *per* dosage leads to a decrease in period solely when driven from the endogenous promoter; instead, when driven from a heterologous and constitutive promoter, it results in a period increase [41],[59]–[61]. This particular situation allows testing genetic interactions with clock genes regarding period determination.

In this context, the strong genetic interaction between *shn* and the core clock components *per*, *tim*, and *Clk* (Figures 6, 7, and S6) prompted us to ask whether modulation of the endogenous period, through activation of the BMP pathway, could result from the regulation of those genes at the transcriptional level. Interestingly, *shn* overexpression reduced *per* and *tim* transcription (Figure 6). More importantly, the activation of the BMP pathway in the sLNvs is associated with reduced *Clk* transcription and protein accumulation, potentially through direct regulation on the DNA binding elements found within the *Clk* promoter region (Figures 7 and S6).

Early on it was suggested that CLK is key to define the amplitude of the rhythms, although the period (and hence the phase) depended on PER and TIM [62]; moreover, sustained and elevated CLK has only minor effects on the periodicity of behavioral rhythms [63]. On the other hand, CLK embodies the rate-limiting step in these molecular oscillations [64], and thus it would be unexpected to see no effect on overt rhythmicity resulting from alterations of CLK levels. Accordingly, it was recently shown that modulating VRI effects on *Clk* transcription through the α isoform of *kayak* (*kay-*α, the mammalian homolog of *c*-*fos*) impinges upon period determination [65]. Interestingly, reduced *kay-*α correlates with lengthening of the behavioral period and reduction in PER, PDP1, and CLK levels in the sLNvs, underscoring that altered CLK levels could indeed affect the pace of the clock.

### Retrograde Signaling as a Network Coordination Mechanism

Previous studies have extensively described the interconnected negative feedback loops critical for the cell-autonomous setting of the 24-h endogenous period, as well as the role of the sLNvs in this process [1]. However, the ability of the sLNvs to integrate information from the rest of the circadian network is poorly understood. Our findings provide genetic, behavioral, and molecular evidence for a model in which the retrograde BMP signaling cascade in adult circadian pacemaker neurons integrates information from the circadian network to modulate the period of locomotor behavior (Figure 8). It follows that ligands released from yet unidentified sLNv postsynaptic cells are capable of modulating *Clk* transcription, thus contributing to set the endogenous period in a non-cell-autonomous fashion. We propose that the endogenous circadian period results from the action of the molecular clock at the cellular level that depends on the abundance of specific clock proteins, whose transcriptional regulation is modulated by the BMP pathway.

In recent years it has become clear that environmental conditions unevenly affect the properties of subsets of circadian neurons, thus impinging upon their relative hierarchy within the network to achieve a coherent response (reviewed in [66]). In this article we show that the BMP pathway is active in specific subsets of circadian neurons (Figures 2 and S2), which may or not coincide with those releasing specific BMP ligands (Figure 3). Although it is too early to ascribe a function to this signaling pathway within the circadian network, it is tempting to speculate that it might provide a means to alter the connectivity of the circuit to keep it tuned to changes in day length or temperature fluctuations, often experienced under natural conditions.

On the other hand, this pathway could also be functional to adjust the endogenous period in response to internal stimuli. In flies it has been proposed that different subsets of circadian neurons run with different periods (for example, [67],[68]). A network capable of sustaining rhythmicity after several days under free-running conditions ensures that several mechanisms should be in place to guarantee coherence between clusters.

The circadian system in multicellular organisms relies on both cell autonomous biochemical interactions and network assembly of specific clusters [69]. The existence of retrograde signals capable of retrieving information from distinct circadian clusters, which then impinge upon core pacemaker properties, offers a provocative hypothesis worth exploring in other circadian networks. Indeed, the BMP signal transduction pathway is highly conserved between invertebrates and vertebrates [19]. In the mammalian system, the suprachiasmatic nucleus (SCN) is composed of a heterogeneous network of coupled oscillators; a retrograde communication between them through the BMP pathway could arise as a novel mechanism involved in synchronization of SCN activity.

## Materials and Methods

### Fly Stocks

Flies were maintained at 25°C on standard fly food on a 12∶12 h LD regimen, except in the indicated experiments. The following strains were used in this study: *w^1118^* (as wild type), *pdf*GAL4, *tim*GAL4, *per*GAL4, *tub*GAL80^ts^, *heatshock*GAL4, UAS-*gfp^nls^*, UAS-*cd8gfp*, UAS-*cd8rfp*, UAS-*rfp^myr^*, UAS-*shn* ^RNAi^ (34689), UAS-*act* ^RNAi^ (29597), UAS-*dpp* ^RNAi^ (33618), UAS-*myo* ^RNAi^ (31200), UAS-*gbb* ^RNAi^ (34898), UAS-*mav* ^RNAi^ (34650), UAS-*med* ^RNAi^ (31928), *dad*LacZ (stock number 10305), all from the Bloomington Stock Center; UAS-*wit* ^RNAi^ (transformant ID 103808), UAS-*sax* ^RNAi^ (transformant ID 46358), UAS-*tkv* ^RNAi^ (transformant ID 105834), UAS-*scw* ^RNAi^ (105303), UAS-*daw* ^RNAi^ (105309), UAS-*mad* ^RNAi^ (110715), and UAS-*dicer2* from the Vienna *Drosophila* RNAi Center; *ClkG* [43] and *pdf*GAL80 [8] from M. Rosbash (Brandeis University); UAS-*shn* [30] from M. Affolter (Basel University), which was only employed in Figures 3 and S1D,E; UAS-*tkv* ^QD^ [33], referred in this work as UAS-*tkv* ^A^ for simplicity, and UAS-*sax* ^A^ [32] from K. Wharthon (Brown University); UAS-*per*3.2 [41], UAS-*tim*3.1 from A. Sehgal (University of Pennsylvania); UAS-*Clk* from R. Allada (Northwestern University); *per*LUC (BG-LUC) [70] and *tim*LUC [71] from Ralf Stanewsky; and *Clk^6936^*G4 and *Clk^856^*G4 [51] from N. Glossop (University of Manchester).

### Locomotor Activity Assay

The locomotor activity of individual male flies was measured using *Drosophila* Activity Monitors (Trikinetics) and analyzed employing ClockLab analysis software (Actimetrics) [72]. Briefly, flies were entrained to 12 h LD cycles during their entire development, and newly eclosed adult males were placed in glass tubes containing standard food. Activity was monitored in LD conditions for 3–4 d, followed by constant darkness for 9 d. Flies with a single peak over the significance line (*p*<0.05) in a chi-square analysis were scored as rhythmic, which was confirmed by visual inspection of the actograms. Flies classified as weakly rhythmic as previously described [73] were not taken into account for average period calculations. A minimum of three independent experiments including 20–32 flies per genotype were analyzed. Fast Fourier Transformation (FFT) analysis for a 24 h period was also carried out with the ClockLab analysis software.

Period and rhythmicity analysis were conducted by one-way ANOVA followed by Tukey comparisons using α = 0.05.

### Immunohistochemistry

Brains were fixed in 4% paraformaldehyde in PB (100 mM KH_2_PO_4_,/Na_2_HPO_4_) and then rinsed three times in PT (PBS supplemented with 0.1% Triton X-100). Brains were then blocked in 7% goat serum in PT for 1 h at room temperature (RT). After the blocking step, tissue was incubated with primary antibodies ON at 4°C. The primary antibodies used were chicken anti-GFP (1/500, Upstate), rat anti-PDF (1/500, custom-made, [74], rabbit anti-RFP (1/1,000, Rockland, USA), rat anti-LacZ (1/500), rabbit anti-P.MAD (1/500 Smad3 Phospho p(S423/425) Epitomics, US), rabbit anti-PER (R. Stanewsky, University College, London), and guinea pig anti-CLK (P. Hardin, Texas A&M University, Texas). The secondary antibodies used were Cy2-conjugated donkey anti-chicken, Cy3-conjugated donkey anti-guinea pig, Cy3-conjugated donkey anti-rabbit, and Cy5-conjugated donkey anti rat (Jacksons Immunoresearch) diluted to a final concentration of 1/250, incubated for 2 h at RT. After staining, brains were washed three times for 15 min and mounted in 80% glycerol (in PT).

### Confocal Analysis and Quantitation

To visualize clock neurons, optical sections of whole brains were taken using a Zeiss LSM510 confocal microscope (Carl Zeiss, Thornwood, NJ). Confocal images were analyzed employing the ImageJ software (NIH).

For time course analysis, quantitation of PER nuclear immunoreactivity was conducted from images derived from single confocal planes. For a given time course, all pictures were taken employing the same confocal settings. The sLNvs were identified by means of the PDF immunoreactivity and their size. The position of the nucleus was determined by the GFP^nls^ signal. For each small LNvs cell body, the mean pixel intensity of PER staining was determined in the nucleus. Each value represents an average of the mean intensity of 2–4 sLNvs per brain and 9–10 brains were analyzed in each experiment.

For transcription activity assays, quantitation of GFP nuclear immunoreactivity was evaluated identical to PER measurement and was scored blindly. Quantitation of CLK nuclear immunoreactivity was carried out as previously described for PER and scored blindly.

To quantify the maximum length of the sLNvs axonal projections at the dorsal protocerebrum, an adaptation of the Sholl method [75] was used. Ten brains were analyzed in each experiment. For each brain a confocal stack including the entire dorsal arborization was obtained and the images were projected to a single plane with a maximum intensity algorithm. The last ring reached by the longest projection was used as an indicator of the overall structure. Whenever possible the projection of the hemisphere on the right side was taken into account for the analysis.

### Luciferase Assays

Luciferase activity in head extracts was measured by using a commercially available assay kit (Promega, USA). Five- to 8-d-old flies were placed on ice and decapitated in 500 µl of homogenization buffer (Passive Lysis Buffer -PLB-) at ZT02; heads were grinded in 200 µl of PLB and centrifuged at 9,279 g at 4°C. The supernatant was subjected to a second round of centrifugation and kept at −80°C. Thirty µl of the resulting extract were incubated for 1 min at room temperature with 100 µl of LARII buffer. Bioluminescence was measured using white plates in a Veritas Microplate Luminometer (Turner BioSystems). Two aliquots of each sample were assayed, and the average of the two readings was taken as the light activity of each sample. The luciferase activity was relativized to the total protein levels assessed by the absorbance at 280 nm and then normalized to the mean luciferase activity of each experiment in order to avoid the impact of different activity assessments due to technical errors. Three independent samples were obtained.

### Identification of MAD and MED Binding Sites

MAD/MED binding sites were scanned as independent strings within *Clk* regulatory region [51]. Then, locations for both binding sites close to each other were analyzed in-depth for orientation and spacing to be compatible with simultaneous binding of both proteins. When both binding sites overlapped (GCCGTCTG), the complex was assumed not to occur due to steric hindrance. We found several binding sites for either MAD or MED but only one location in which sites for both proteins are close to each other. Sites are spaced by 9 and 21 base pairs. Even though both spacers are longer than the 5 bp reported, we hypothesize that 21 (5+16) would allow both sites to be displayed in the same side of the DNA molecule, one turn and a half away and close enough to interact. *Clk* regulatory sequences were obtained from FlyBase (FBgn0023076).

### Heat Shock Treatment

Just before the procedure flies were transferred to an empty glass vial (with no food). Two heat shock (37°C, 30 min each) pulses were delivered in a water bath followed by recovery periods (3 and 2 h long, respectively). RNA extraction was carried out after the 6 h treatment.

### Real-Time PCR

Total RNA isolation from fly head extracts was performed using Trizol (Invitrogen, Carlsbad, CA); SuperScript II (Invitrogen) was used for reverse transcription following the manufacturer's instructions.

The real-time assays were conducted with the Stratagene Mx3000P QPCR System (La Jolla, CA) using FastStart Universal SYBR Green Master (ROX) from Roche. *tubulin* was used as the reference gene. The primers were designed using Primer3 (available online at http://frodo.wi.mit.edu/primer3/). The primers employed to measure *shn* transcripts were: 1-shnA/DF2:TTCACGCAAGAGTGCTTTGGAAACG; 2-shnBF1:CGGGCCGCAATATCTCTCAGATTAGT; 3-shnCF1:TTCGATTTCTTGCTATTCGCGCCG; 4-shnDF1:AACGGGACACCAACTTTGAAGCAG; 5-shnF1E8:GTGCAGCAACCGGATGTCAATGAA; 6-shnR3:GGCACGTCGCGTATTGTTCACTT. The primers used to amplify *tubulin* were: tubF GCCTGAACATAGCGGTGAAC and tubR ATCCCCAACAACGTGAAGAC.

### Statistical Analysis

Statistical analyses were performed with InfoStat version 2009 (Grupo InfoStat, FCA, Universidad Nacional de Córdoba, Argentina). The statistical analysis carried out in each experiment is described in the corresponding figure legend.

## Supporting Information

Figure S1 **The long period phenotype is due to** ***shn*** **overexpression.** (A) Schematic diagram of the *shn* locus displaying the four alternative transcription initiation sites, the relative position of the transposon, and the primers employed in Figure S1B and S1C. The numbers in the diagram refer to the primers employed (the sequence is listed in the Materials and Methods section). (B) Quantitation of *shn* alternative variants in homozygous or heterozygous P[UAS]^756^ flies relative to *w^1118^*. (C) Quantitation of *shn* alternative variants in pulsed *heatshock*G4> P[UAS]^756^ flies relative to nonpulsed siblings. (D and F) Representative double-plotted actograms of flies of the indicated genotypes. Locomotor behavior was monitored as explained in the legend to Figure 1E. (E and G) Graphs show the quantitation of period and rhythmicity for the indicated genotypes (see legend to Figure 1C for more details). Statistical analysis included a one-way ANOVA for period determination; different letters indicate a significant difference in Tukey comparisons, α = 0.05. Error bars represent SEM, and averages represent at least three independent experiments.
(EPS)

Figure S2 **BMP pathway activation pattern in adult circadian neurons.** (A and B) Spatial expression of DAD is visualized through a LacZ reporter (red), while clock cells are identified by immunostaining with anti-PER antibody (green). The BMP pathway is absent from the LNds, clearly present in the small and large LNvs (A), and also in a few DN1s cells (B). Scale bar, 10 µm.
(EPS)

Figure S3 **Circadian period lengthening requires** ***tkv*** **and** ***sax*** **activation.** (A) Whole mount brain immunofluorescence was performed to follow PDF (upper and lower panels) and pMAD (middle and bottom panels) accumulation at ZT02 in the indicated genotypes. The circadian period for locomotor behavior of each genotype is indicated (see also Figure 2). Scale bar, 5 µm. (B) Quantitation of pMAD nuclear intensity. PDF+ staining and cell body size were used to identify the sLNvs. From 8 to 10 brains were analyzed in each experiment; the average of 2–4 neurons was used for each determination, and the images were taken employing the same confocal settings throughout an individual experiment. pMAD was not detectable in the *w^1118^* and *pdf*>*sax* ^A^ flies, so these genotypes were excluded from the analysis. Three independent experiments were carried out, and data were analyzed with Student's *t* test.
(EPS)

Figure S4 **Adult-specific** ***shn*** **overexpression does not correlate with structural defects.** (A) Schematic diagram of the experiment shown in Figure S4B and S4C; flies were raised at 25°C under LD cycles; 2–5-d-old flies were kept at 25°C for 3 additional days. Then they were transferred to 30°C and kept in LD cycles in order to avoid accumulating differences due to the distinct endogenous periods. *shn* expression was induced for 2 or 9 d, and the brains were analyzed by immunohistochemistry at ZT2. (B and C) Representative images of whole brains stained with anti-RFP after 9 d of *shn* induction. (B) Confocal stacks of a brain hemisphere are shown. (C) Confocal stacks of sLNvs dorsal projections including the concentric rings used for quantitation. (D) Quantitation of the average maximum ring reached by the sLNv projections in brains overexpressing *shn* for 2 or 9 d under LD conditions. No significant differences were observed. (E) Schematic diagram of the experiment shown in Figure S4F and S4G (see legend to Figure 4A for more details). (F) Representative double-plotted actograms of flies of the indicated genotypes. The blue and red shadows represent the phase at the restricted (25°C) and permissive (30°C) temperature, respectively. Gray arrows indicate the transfer to constant darkness. (G) Bar diagram shows the difference between the endogenous period at the permissive and restrictive temperatures for each specific genotype. Data were analyzed with a Student's *t* test, * *p* = 0.004. Error bars represent SEM, and averages represent at least three independent experiments.
(EPS)

Figure S5 ***pdf*** **G4>** ***shn*** **behavioral phenotype is independent of the number of UAS driven transgenes.** (A) Representative double-plotted actograms of flies of the indicated genotypes. Locomotor behavior was monitored as explained in the legend to Figure 1E. (B) The graph shows the quantitation of period and rhythmicity for the indicated genotypes (see legend to Figure 1C for more details). Statistical analysis included a one-way ANOVA for period determination; different letters indicate a significant difference in Tukey comparisons, α = 0.05. Error bars represent SEM, and averages represent at least three independent experiments.
(EPS)

Figure S6 **BMP activation modulates** ***Clk*** **promoter activity.** (A) Representative double-plotted actograms of flies of the indicated genotypes. Behavioral experiments were carried out as detailed in the legend to Figure 1E. (B) Graph shows the quantitation of period and rhythmicity for the indicated genotypes (see legend to Figure 1C for more details). Statistical analysis included one-way ANOVA for period determination, and different letters indicate significant differences in Tukey comparisons, α = 0.05. Error bars represent SEM, and averages represent at least three independent experiments. (C) The schematic diagram depicts the *Clk* locus highlighting the existence of five alternative transcripts (according to FlyBase) and the DNA fragment contained in the GAL4 reporter line; gray boxes represent untranslated exons, red indicates translated exons, and green indicates the fusion between the first 18 amino acids of CLK and the transcription factor GAL4 (not to scale). Putative MAD (red) and MED (blue) binding elements, and the relative position within the locus are indicated. The CLK^6939^GAL4 reporter line contains a total of 8,417 bp and starts 1,660 bp upstream of the transcription starting point. (D) Whole mount brain immunofluorescence was performed to follow PDF (upper panel) and GFP (upper and bottom panels) accumulation at ZT2 in the −6,939 *Clk* promoter reporter (*Clk* ^6939^, [51]). (E) Quantitation of GFP nuclear intensity. PDF+ staining and cell body size were used to identify the sLNvs. From 8 to 10 brains were analyzed in each experiment; the average of 2–4 neurons was used for each determination, and the images were taken employing the same confocal settings throughout an individual experiment. Three independent experiments were carried out. Data were analyzed with Randomized Blocks ANOVA to contemplate potential differences due to the different confocal settings. Paired measurements are linked by colored lines. Pblocks = 0.55, pgenotypes = 0.04.
(EPS)

Table S1 **Behavioral analysis; quantitation of period and rhythmicity.**
(DOCX)

Table S2 **Blocking BMP signaling severely alters circadian behavior in constant conditions.**
(DOCX)

Table S3 **BMP ligands have a complex effect on circadian neural clusters.**
(DOC)

## Abbreviations

ANOVA: analysis of variance
BMP: bone morphogenetic protein
CNS: central nervous system
CT: circadian time
*dad*: *daughters against dpp*
*dClk*: *Drosophila Clock*
DD: constant darkness
DN1: dorsal neurons type 1
DNA: Deoxyribonucleic acid
*gbb*: *glass bottom boat*
GFP: Green Fluorescent Protein
LD: light∶dark
LNd: dorsal lateral neurons
LUC: luciferase
*mad*: *mothers against dpp*
*med*: *medea*
nls: nuclear localization signal
NMJ: neuromuscular junction
ORFs: open reading frames
PDF: PIGMENT DISPERSING FACTOR
PDP1e: PAR-domain protein 1ε
*per*: *period*
pMad: phosphorylated mothers against dpp
*put*: *punt*
qPCR: quantitative polymerase chain reaction
RFP: red fluorescent protein
RNAi: RNA interference
*sax*: *saxophone*
SCN: suprachiasmatic nucleus
SEM: standard error of the mean
*shn*: *schnurri*
sLNvs: small lateral ventral neurons
TARGET: temporal and regional gene expression targeting
*tim*: *timeless*
*tkv*: *thickveins*
UAS: upstream activating sequence
*vri*: *vrille*
*wit*: *wishful thinking*
ZT: zeitgeber time
