# Supplementary material for: Circadian Period Integrates Network Information Through Activation of the BMP Signaling Pathway
Source: PLoS Biol. 2013 Dec 10;11(12):e1001733. doi: 10.1371/journal.pbio.1001733 (PMC3858370; doi:10.1371/journal.pbio.1001733)
Supplement: Table S1 — Behavioral analysis; quantitation of period and rhythmicity. (DOCX) [file pbio.1001733.s007.docx]

Table S1. Behavioral analysis; quantitation of period and rhythmicity.

|  | Genotype | N | τ | τ SEM | %R | %R SEM |
| --- | --- | --- | --- | --- | --- | --- |
| *shn* overexpresion in the circadian network | | | | | | |
|  | P[UAS]^756^/+ | 3 | 24.0 | 0.01 | 91.0 | 4.0 |
|  | P[UAS]^756^/P[UAS]^756^ | 3 | 23.9 | 0.02 | 73.3 | 10.0 |
|  | *pdf*G4>*cd8gfp* | 3 | 24.1 | 0.21 | 85.6 | 6.2 |
|  | *tim*G4>*cd8gfp* | 3 | 23.9 | 0.23 | 82.0 | 9.2 |
|  | *tim*G4,*pdf*G80 | 3 | 24.0 | 0.28 | 89.3 | 7.6 |
|  | *pdf*G4>P[UAS]^756^ | 4 | 25.3 | 0.12 | 91.8 | 2.2 |
|  | *tim*G4>P[UAS]^756^ | 4 | 25.8 | 0.33 | 81.2 | 5.3 |
|  | *tim*G4,*pdf*G80>P[UAS]^756^ | 3 | 23.8 | 0.28 | 83.3 | 12.3 |
| *shn* downregulation | | | | | | |
|  | *pdf*G4>*dicer2*,*cd8gfp* | 3 | 24.2 | 0.23 | 92.0 | 1.7 |
|  | *shn*^RNAi^/+ | 3 | 24.1 | 0.12 | 100.0 | 0.0 |
|  | *pdf*G4>*dicer2*,*shn*^RNAi^ | 3 | 24.3 | 0.29 | 49.1 | 0.9 |
| BMP pathway activation | | | | | | |
|  | *pdf*G4> *cd8gfp* | 3 | 24.2 | 0.25 | 95.5 | 0.3 |
|  | *pdf*G4>*tkv^A^*;*sax^A^* | 3 | 27.4 | 0.09 | 96.8 | 0.5 |
|  | *pdf*G4>*tkv^A^* | 3 | 24.0 | 0.00 | 97.0 | 0.3 |
|  | *pdf*G4>*sax^A^* | 3 | 24.0 | 0.04 | 98.5 | 0.3 |
|  | *tkv^A^* /+ | 3 | 23.7 | 0.06 | 96.9 | 0.5 |
|  | *sax^A^* /+ | 3 | 23.5 | 0.27 | 100.0 | 0.0 |
| BMP pathway downregulation | | | | | | |
|  | *tkv*^RNAi^/+ | 4 | 23.8 | 0.10 | 96.8 | 2.1 |
|  | *pdf*G4>*dicer2*,*tkv*^RNAi^ | 4 | 23.9 | 0.17 | 62.1 | 3.3 |
|  | *sax*^RNAi^/+ | 4 | 23.8 | 0.11 | 97.0 | 1.7 |
|  | *pdf*G4>*dicer2*,*sax*^RNAi^ | 4 | 24.3 | 0.34 | 59.4 | 9.0 |
|  | *wit*^RNAi^/+ | 4 | 23.8 | 0.15 | 100.0 | 0.0 |
|  | *pdf*G4>*dicer2*,*wit*^RNAi^ | 4 | 24.1 | 0.19 | 70.8 | 7.5 |
| BMP nuclear elements downregulation | | | | | | |
|  | *pdf*G4>*dicer2*,*cd8gfp* | 3 | 24.7 | 0.14 | 89.3 | 1.3 |
|  | *mad^RNAi^,med*^RNAi^/+ | 3 | 23.7 | 0.12 | 92.2 | 7.6 |
|  | *pdfG4>dicer2,mad^RNAi^;med*^RNAi^ | 3 | 24.2 | 0.10 | 34.2 | 13.5 |
| *tim* and *per* rescue | | | | | | |
|  | *pdf*G4>*cd8gfp* | 3 | 24.0 | 0.10 | 97.0 | 1.6 |
|  | *pdf*gal4>shn | 3 | 25.4 | 0.23 | 93.8 | 4.8 |
|  | uasPER/+ | 3 | 23.8 | 0.12 | 100.0 | 0.0 |
|  | *pdf*gal4/uasPER | 2 | 25.2 | 0.09 | 67.8 | 2.2 |
|  | *pdf*gal4,uasSHN/uasPER | 3 | 24.4 | 0.10 | 95.6 | 2.9 |
|  | uasTIM/+ | 3 | 23.6 | 0.06 | 98.0 | 2.0 |
|  | *pdf*gal4/uasTIM | 3 | 24.1 | 0.12 | 57.8 | 16.5 |
|  | *pdf*gal4,uasSHN/uasTIM | 3 | 24.1 | 0.16 | 61.7 | 8.3 |
| *clk* genomic rescue | | | | | | |
|  | *pdf*G4>*cd8rfp* | 4 | 24.4 | 0.03 | 85.9 | 6.2 |
|  | *pdf*G4>P[UAS]^756^,*cd8rfp* | 4 | 25.6 | 0.36 | 85.4 | 10.7 |
|  | *pdf*G4>*dCLK* | 5 | 23.6 | 0.08 | 95.3 | 2.1 |
|  | *dCLK*/+ | 5 | 23.4 | 0.14 | 98.0 | 0.8 |
|  | *pdf*G4>P[UAS]^756^,*dCLK* | 4 | 24.8 | 0.18 | 87.1 | 4.3 |

N: number of experiment analyzed, 20-32 animals were analyzed in each experiment

τ indicates the average endogenous period in constant conditions.

%R indicates percent flies with detectable rhythmicity (see Methods for details).

SEM: standard error of the mean.
