# Supplementary material for: Circadian Period Integrates Network Information Through Activation of the BMP Signaling Pathway
Source: PLoS Biol. 2013 Dec 10;11(12):e1001733. doi: 10.1371/journal.pbio.1001733 (PMC3858370; doi:10.1371/journal.pbio.1001733)
Supplement: Table S2 — Blocking BMP signaling severely alters circadian behavior in constant conditions. (DOCX) [file pbio.1001733.s008.docx]

Table S2 Blocking BMP signaling severely alters circadian behavior in constant conditions.

| Genotype | Normalized FFT | | | |
| --- | --- | --- | --- | --- |
|  | Mean | SEM | N | p value |
| *shn*^RNAi^/+ | 1,206 | 0,218 | 3 | 0,358 |
| *pdf*G4>*dicer2*;*shn*^RNAi^ | 0,919 | 0,260 | 3 |  |
| *tkv*^RNAi^/+ | 1,056 | 0,054 | 4 | 0,016 |
| *pdf*G4>*dicer2*; *tkv*^RNAi^ | 0,823 | 0,061 | 4 |  |
| *sax*^RNAi^/+ | 1,245 | 0,106 | 4 | 0,060 |
| *pdf*G4>*dicer2*; *sax*^RNAi^ | 0,986 | 0,074 | 4 |  |
| *wit*^RNAi^/+ | 1,152 | 0,049 | 4 | 0,042 |
| *pdf*G4>*dicer2*; *wit*^RNAi^ | 0,941 | 0,081 | 4 |  |
| *mad*^RNAi^;*med*^RNAi^/+ | 1,374 | 0,149 | 3 | 0,072 |
| *pdf*G4>*dicer2*; *mad*^RNAi^;*med*^RNAi^ | 0,968 | 0,139 | 3 |  |

Table 2 details the behavioral analysis of rhythmic flies evaluated in the experiments shown in Figure 1E, Figure 2E and Figure 2G under DD. The subset of rhythmic flies among those with downregulated expression of specific elements in the pathway show lower FFT peak compared to rhythmic controls, arguing that despite being classified as rhythmic, downregulated flies show lower amplitude rhythms. N indicates number of experiments analyzed, and SEM indicates the standard error of the mean.
