# Supplementary material for: Circadian Period Integrates Network Information Through Activation of the BMP Signaling Pathway
Source: PLoS Biol. 2013 Dec 10;11(12):e1001733. doi: 10.1371/journal.pbio.1001733 (PMC3858370; doi:10.1371/journal.pbio.1001733)
Supplement: Table S3 — BMP ligands have a complex effect on circadian neural clusters. (DOC) [file pbio.1001733.s009.doc]

Table S3. BMP ligands have a complex effect on circadian neural clusters.

| Genotype | N | τ | τ SEM | %R | %R SEM | FFT | FFT SEM |  | Stats |  |  |  |  |  |  |
| --- | --- | --- | --- | --- | --- | --- | --- | --- | --- | --- | --- | --- | --- | --- | --- |
| *pdf*G4>*dicer2* | 8 | 24.1 | 0.05 | 95.4 | 2.3 | 0.072 | 0.010 |  | d |  |  |  |  |  |  |
| *tim*G4,*pdf*G80>*dicer2* | 8 | 23.9 | 0.06 | 93.2 | 4.4 | 0.062 | 0.007 |  | c,d |  |  |  |  |  |  |
| *myo*^RNAi^/+ | 3 | 23.6 | 0.05 | 97.8 | 2.7 | 0.063 | 0.004 |  |  |  |  |  |  |  |  |
| *pdf*G4>*dicer2,myo*^RNAi^ | 3 | 24.1 | 0.06 | 50.6 | 15.8 | 0.034 | 0.002 |  | a,b,c |  |  |  |  |  |  |
| *tim*G4,*pdf*G80>*dicer2,myo*^RNAi^ | 3 | 23.7 | 0.20 | 61.0 | 21.1 | 0.038 | 0.008 |  | a,b,c |  |  |  |  |  |  |
| *gbb*^RNAi^/+ | 4 | 23.7 | 0.16 | 98.4 | 1.8 | 0.058 | 0.005 |  |  |  |  |  |  |  |  |
| *pdf*G4>*dicer2,gbb*^RNAi^ | 4 | 24.1 | 0.12 | 42.7 | 5.8 | 0.032 | 0.007 |  | a,b,c |  |  |  |  |  |  |
| *tim*G4,*pdf*G80>*dicer2,gbb*^RNAi^ | 4 | 23.5 | 0.06 | 66.3 | 12.1 | 0.032 | 0.005 |  | a,b |  |  |  |  |  |  |
| *mav*^RNAi^/+ | 4 | 23.7 | 0.14 | 93.8 | 5.1 | 0.048 | 0.006 |  |  |  |  |  |  |  |  |
| *pdf*G4>*dicer2,mav*^RNAi^ | 4 | 23.9 | 0.08 | 27.1 | 8.0 | 0.028 | 0.005 |  | a,b |  |  |  |  |  |  |
| *tim*G4,*pdf*G80>*dicer2,mav*^RNAi^ | 4 | 23.5 | 0.09 | 83.3 | 2.0 | 0.029 | 0.003 |  | a,b |  |  |  |  |  |  |
| *dpp*^RNAi^/+ | 3 | 23.7 | 0.15 | 93.6 | 4.4 | 0.038 | 0.004 |  |  |  |  |  |  |  |  |
| *pdf*G4>*dicer2,dpp*^RNAi^ | 3 | 24.0 | 0.09 | 54.9 | 11.3 | 0.031 | 0.002 |  | a,b |  |  |  |  |  |  |
| *tim*G4,*pdf*G80>*dicer2,dpp*^RNAi^ | 3 | 23.5 | 0.16 | 72.3 | 10.5 | 0.022 | 0.005 |  | a |  |  |  |  |  |  |
| *act*^RNAi^/+ | 6 | 23.6 | 0.08 | 94.6 | 3.2 | 0.056 | 0.011 |  |  |  |  |  |  |  |  |
| *pdf*G4>*dicer2,act*^RNAi^ | 6 | 23.9 | 0.07 | 73.2 | 8.2 | 0.034 | 0.004 |  | a,b |  |  |  |  |  |  |
| *tim*G4,*pdf*G80>*dicer2,act*^RNAi^ | 6 | 23.5 | 0.11 | 63.6 | 9.1 | 0.033 | 0.008 |  | a,b,c |  |  |  |  |  |  |
| *daw*^RNAi^/+ | 4 | 23.5 | 0.02 | 98.4 | 1.8 | 0.078 | 0.009 |  |  |  |  |  |  |  |  |
| *pdf*G4>*dicer2,daw*^RNAi^ | 4 | 24.3 | 0.10 | 71.0 | 11.0 | 0.048 | 0.005 |  | a,b,c,d |  |  |  |  |  |  |
| *tim*G4,*pdf*G80>*dicer,2daw*^RNAi^ | 4 | 23.5 | 0.11 | 97.5 | 2.9 | 0.062 | 0.004 |  | c,d |  |  |  |  |  |  |
| *scw*^RNAi^/+ | 3 | 23.6 | 0.05 | 95.8 | 2.6 | 0.074 | 0.010 |  |  |  |  |  |  |  |  |
| *pdf*G4>*dicer2,scw*^RNAi^ | 3 | 24.0 | 0.06 | 73.8 | 5.2 | 0.037 | 0.006 |  | a,b,c |  |  |  |  |  |  |
| *tim*G4,*pdf*G80>*dicer2,scw*^RNAi^ | 3 | 23.6 | 0.05 | 100.0 | 0.0 | 0.056 | 0.007 |  | b,c,d |  |  |  |  |  |  |

N indicates number of experiment analyzed, 10-16 animals were analyzed in each experiment.

τ indicates the average endogenous period in constant conditions.

%R indicates percent flies with detectable rhythmicity (see Materials and Methods for details).

FFT indicates the Fast Fourier Transformation for a 24h period

SEM: standard error of the mean.

Stats. Statistical analysis included one way ANOVA; the columns labeled with different letters (a, b, c) indicate significantly different treatments in Tukey comparisons, α=0.05. To simplify the statistical analysis the heterozygous RNAi insertions (ligand^RNAi^/+), which are highly rhythmic, were not included.
